# Optimizing the use of acoustic materials in office buildings

**DOI:** 10.1038/s41598-021-00082-3

**Published:** 2021-10-19

**Authors:** Abdullah AlOmani, Khaled El-Rayes, Ayman Altuwaim

**Affiliations:** 1 Department of Civil and Environmental Engineering, University of Illinois at Urbana-Champaign, Urbana, IL 61801 USA; 2 Department of Civil Engineering, College of Engineering, King Saud University, Riyadh, Saudi Arabia

**Keywords:** Civil engineering, Engineering, Mechanical engineering

## Abstract

Office space designers encounter a challenge in identifying the optimal set of noise control materials to improve the acoustic quality while keeping the cost of selected acoustic materials to a minimum. To address this challenge, this paper presents a novel optimization model that provides the capability of minimizing the cost of acoustic materials while satisfying all designer-specified acoustic quality requirements. The model is developed in five main stages that focus on (1) identifying the correlated designer decisions that influence the model objective function; (2) formulating an optimization objective function; (3) identifying the model constraints that are organized into acoustic quality and materials selection constraints; (4) implementing the model using genetic algorithms (GA); and (5) evaluating the performance of the model using an office space design that is under construction to assess and improve the model feasibility and performance. The outcome of the performance evaluation stage illustrates the novel capabilities of the developed model in identifying the optimal selections for the type and area of acoustic material for each surface in the office space that achieve the desired acoustic quality while keeping the cost of selected acoustic materials to a minimum.

## Introduction

A study reported that more than 33 million people working in office buildings in the USA, and the number of these buildings was estimated to be more than one million which represents 18% of the total commercial buildings in the nation^1^. Other studies also reported that (a) people spend half of their waking hours in office spaces which is almost third of their entire life^2^; (b) the comfort and health of office space occupants are significantly affected by the acoustic design of these spaces^3–5^; (c) noise levels in office spaces adversely affect the productivity of their occupants and their job satisfaction^6,7^; and (d) there is an increasing demand for improving acoustic quality in office spaces^8^.

High noise levels in office spaces have been reported to cause human stress, distraction, and low performance in office buildings^6,9–12^, and were also reported to be a major cause of hearing loss if they persisted for a long time^13^. To address these negative impacts, architects, designers, and engineers need to identify and utilize an optimal set of noise control materials in office spaces to minimize these noise levels while keeping their cost to a minimum. The ANSI standard specifies that it is the resposibility of the project designer or archiect to specify the acoustic system and installation methods to achieve the required background noise level in the space^14^. This has been reported to be a challenging task for designers due to the vast number of acoustic materials available with a variety of sound coefficients and costs^15^. To address these acoustic design challenges in office spaces, several research studies focused on improving (1) the noise control design and the acoustic materials coefficient in sound absorption; (2) the performance of sound transmission between adjacent spaces; and (3) the speech intelligibility in the workspace.

The first group of the aforementioned research studies focused on improving the noise control design and sound absorption in office spaces^14,16–21^. For example, a recent study conducted a survey of 237 workers in open-plan offices and reported that the background noise level experienced by these workers was higher than the acceptable level and caused distraction during work time^19^. Other studies reported that office workers irrelevant speech and intelligible conversations in an open-plan office was the most disturbing sound source that needs to be considered in the noise control design^22–24^. Keränen et al. (2008) developed a multivariable regression model to predict and measure the performance of room acoustics in 15 different open offices, and the study reported that room acoustics in open offices can be controlled by increasing sound absorption, installing high screens, and adequate sound insulation. Another study developed a logistic regression model to examine acceptable noise levels in office spaces and reported that the noise level that needs to be maintained in air-conditioned offices is 57.5 dBA^25^. Vervoort and Vercammen (2015) developed a model to predict background noise levels in office spaces including those caused by workers conversation, PC/laptop use, paperwork handling, and writing.

The second group of research studies focused on conducting experiments to improve the performance of sound transmission between two adjacent spaces^24,26–31^. The sound transmission problems between adjoining spaces in office spaces are considered to be one of the primary concerns in architectural acoustics^28^. For example, Kaarlela-Tuomaala et al. (2009) conducted a survey of 31 workers who were moved from private offices to an open-plan office and reported that the speech level of sound transmitted from nearby private offices is significantly lower than that transmitted from neighboring workstations in an open-plan office. Another experiment study investigated sound insulation performance of internal double walls, and reported that stud spacing did not play a significant role except at low frequencies when using dense stud spacing^31^.

The third group of research studies focused on enhancing speech intelligibility in the workspace utilizing a number of experiments and methodologies^16,32–36^. The speech intelligibility indicators define the level of distraction and privacy in office spaces and can be measured using Articulation Index (AI) recommended by ASTM E1130-08, Speech Transmission Index (STI) recommended by standard ISO 3382-3, or Peutz’s formula of Articulation Loss of Consonants ($${\%AL}_{Cons})$$ ^37–41^. For example, Werff and Leeuw (2003) expanded Peutz's formula of (%$${AL}_{Cons})$$ to enhance the prediction of speech intelligibility in buildings by considering sound system design and room acoustic simulation. Wang and Bradley (2002a) developed a model to investigate the acoustic performance in open-plan office space, and reported that ceiling absorption is one of the main factors that significantly enhance speech intelligibility.

Despite the significant contributions of the aforementioned studies, there is no reported research that addressed the aforementioned challenge confronting office space designers who need to identify and utilize optimal set of noise control materials that comply with the specified acoustic quality requirements while keeping the cost of acoustic materials to a minimum. To overcome this limitation, this paper presents a novel model that is capable of optimizing the acoustic design decisions in office spaces to minimize their acoustic materials cost while acheiving the designer-specified acoustic quality requirements.

## Objective

The objective of this research study is to develop a novel model for optimizing the acoustic materials selection of office spaces in order to minimize the total acoustic cost while satisfying all designer-specified acoustic quality requirements. The model is designed to support acousticians, designers, architects, and decision-makers to identify optimal selections of acoustic materials for office spaces. The model is developed in five main stages that focus on (1) identifying all relevant designer decisions that impact the cost of acoustic materials; (2) formulating an objective function that is capable of minimizing the required cost of acoustic materials; (3) identifying all practical constraints in this model that are organized into the following two main groups, acoustic quality constraints and materials selection constraints; (4) implementing the model using genetic algorithms (GA) on MATLAB; and (5) evaluating the performance of the model using a real-world application example of an office space design that is under construction to assess and improve the model feasibility and performance. The present model is designed to comply with all developed practical constraints that use standard acoustic formulas to measure and quantify designer-specified acoustic quality requirements including the equivalent sound absorption, speech intelligibility, and noise reduction level. The following sections will describe in detail these five development stages of the developed model using a real-world application example of office space design.

## Designer decisions

**Figure 1:**
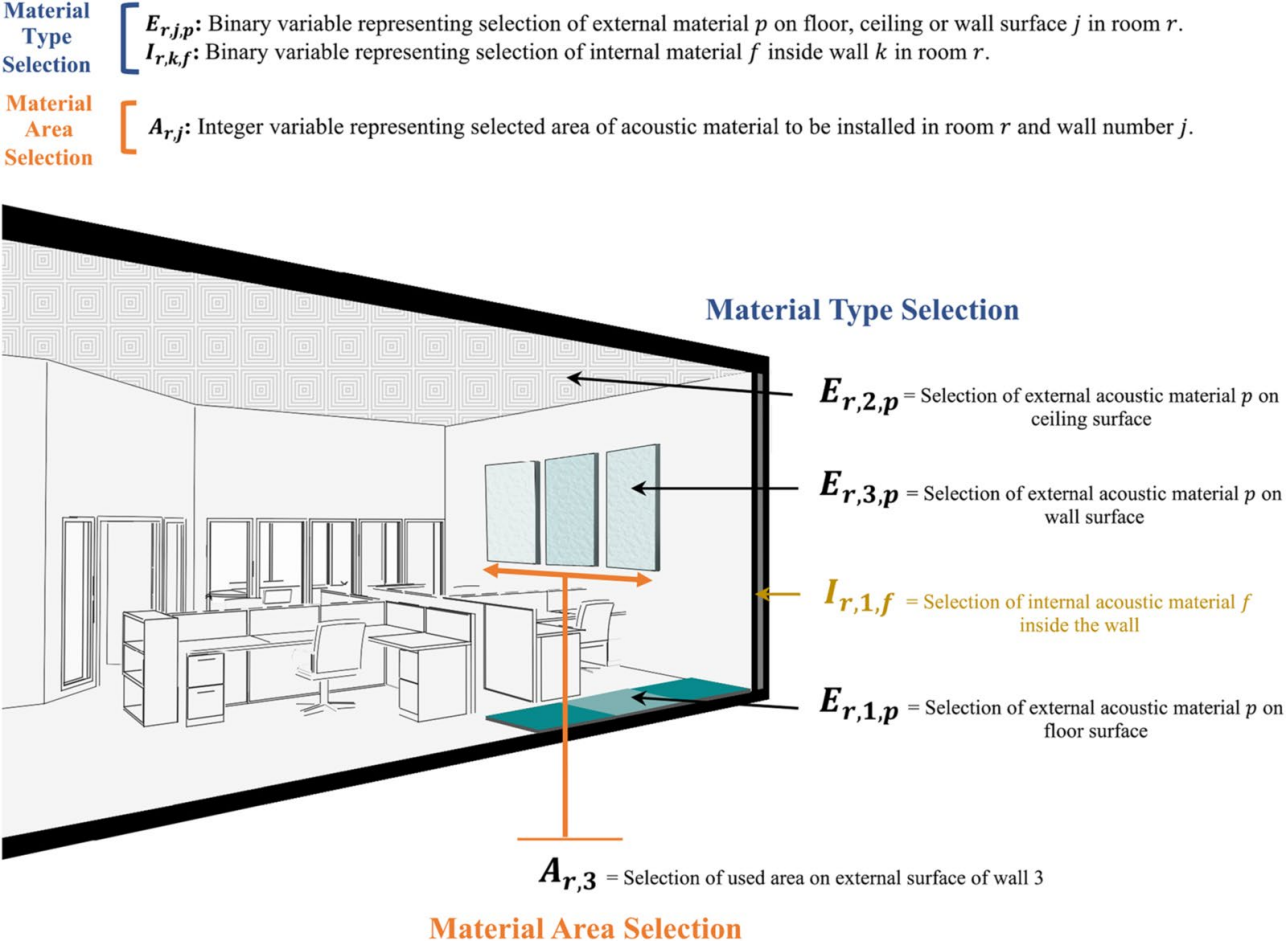
Designer decisions.

The present model incorporates all relevant decisions that designers need to make during the acoustic design of office spaces. The identified designer decisions in this model can be grouped into two main categories that represent the selection of acoustic material type and area. The present model is designed to allow designers to specify the use of two types of acoustic materials in office spaces: (1) external acoustic material (${E}_{r,j,p}$) that is used on the surface of the floor, ceiling, and/or wall; and (2) internal acoustic material (${I}_{r,k,f}$) that is used inside the wall cavity, as shown in Fig. 1. Accordingly, the first category of designer decisions represents the material type selection of external (${E}_{r,j,p}$) and internal (${I}_{r,k,f}$) acoustic material (see Fig. 1). The selection of external acoustic material is modeled using decision (${E}_{r,j,p}$) that represents the designer selection of acoustic material $p$, from a set of feasible alternatives, in order to specify its use on the surface of floor, ceiling, and/or wall $j$ in room $r$. The decision (${E}_{r,j,p}$) is modeled as a binary variable that can have a value of either 1 or 0. A value of 1 for decision (${E}_{r,j,p}$) represents that material $p$ was selected for surface $j$ in room $r$, and (${E}_{r,j,p}$) = 0 represents that material $p$ was not selected for that surface. The selection of internal acoustic material is also modeled using a binary decision (${I}_{r,k,f}$) that represents the selection of acoustic material $f$ inside wall $k$ in room $r$ from a set of feasible alternatives. Where, (${I}_{r,k,f}$) = 1 represents that material $f$ was selected inside wall $k$ in room $r$, and (${I}_{r,k,f}$) = 0 represents that material $f$ was not selected inside wall $k$ in room $r$.

**Figure 2:**
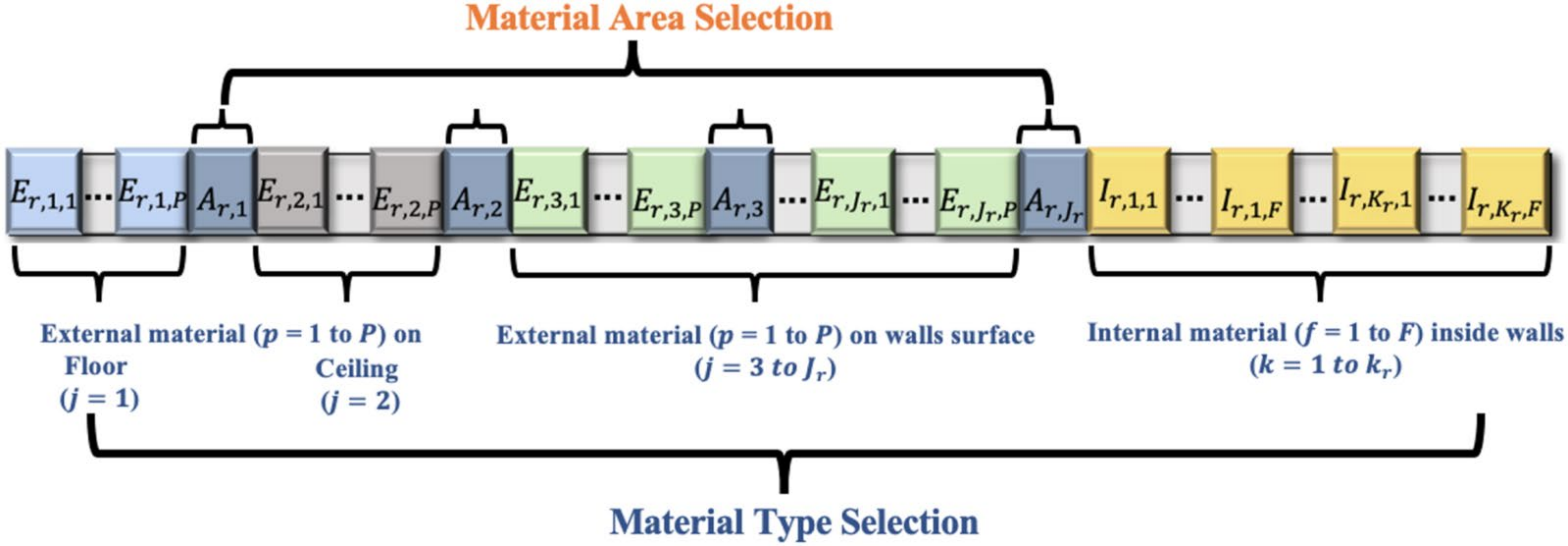
Chromosome example for decision variables.

The second category of designer decisions ($${A}_{r,j})$$ represents the area selection of acoustic material that will be installed on external surface $$j$$ in room $$r$$, as shown in Figs. 1 and 2. Building rooms are modeled using positive integer variable $$r$$, that ranges from 1 to $$R$$. Room surfaces are modeled using positive integer variable $$j$$, where $$j=1$$ represents room floor, $$j=2$$ represents room ceiling, and $$j$$ from 3 to $${J}_{r}$$ represents room wall surfaces, as shown in Figs. 1 and 2.

## Objective function

The present model integrates an objective function that is designed to calculate and minimize the cost of all acoustic materials ($CAM$) in office spaces by adding up all the external and internal acoustic materials costs, as shown in Eq. (1). The external acoustic material cost ($CEM$) is calculated by adding up the unit cost of all selected external acoustic materials multiplied by their selected areas, as shown in Eq. (2). Similarly, the internal acoustic material cost ($CIM$) is calculated by adding up the unit cost of all selected internal acoustic materials multiplied by their areas, as shown in Eq. (3).

1 $${Min}CAM=CEM+CIM$$

2 $$CEM= \sum _{r=1}^{R}\sum_{j=1}^{{J}_{r}}\left(\sum_{p=1}^{P}{C}_{p}*{E}_{r,j,p}*{A}_{r,j}\right)$$

3 $$CIM= \sum _{r=1}^{R}\sum_{k=1}^{{K}_{r}}\left(\sum_{f=1}^{F}{C}_{f}*{I}_{r,k,f}* {S}_{r,k}\right)$$

where $$CAM$$ = total cost of all acoustic materials; $$CEM$$ = total cost of all external acoustic materials;$${C}_{p}$$= unit cost of external acoustic material $$p$$ in $/m^2^; $${E}_{r,j,p}$$= binary decision variable representing the selection of external acoustic material *p* for surface $$j$$ in room $$r$$; $${A}_{r,j}$$= total selected acoustic material area for surface *j* in room *r* in m^2^; $$CIM$$ = total cost of all internal acoustic materials; $${C}_{f}$$= unit cost of internal acoustic material $$f$$ in $/m^2^; $${I}_{r,k,f}$$= binary decision variable representing the selection of internal acoustic material $$f$$ used inside wall $$k$$ in room $$r$$; $$R$$= total number of rooms in the building; $${J}_{r}=$$ total number of wall surfaces in room $$r$$; $$P$$= total number of external acoustic materials that can be installed on floor, ceiling, and/or wall surfaces; $${K}_{r}$$= total number of walls in room $$r$$; $${S}_{r,k}$$= total area of wall $$k$$ in room $$r$$ in m^2^; $$F$$= total number of internal acoustic materials that can be installed inside wall cavities.

## Constraints

The present model is designed to comply with all practical constraints. The identified constraints in this model are organized into the following two main groups: (1) acoustic quality constraints; and (2) materials selection constraints.

### Acoustic quality constraints

#### Sound absorption constraint

The purpose of this constraint is to ensure that the total equivalent sound absorption ${O}_{r}$ in room $r$ is more than or equal its minimum requirement of sound absorption $\ell_{r}$ in room $r$ that comply with the requirements of acoustic standards, as shown in Eq. (4)^38,43–45^. The total equivalent sound absorption ${O}_{r}$ in room $r$ depends on the noise reduction coefficient (NRC) of the aforementioned selection of acoustic materials. The (NRC) is the average of a third-octave band sound absorption coefficients of the particular surface for frequencies of 250 Hz, 500 Hz, 1000 Hz, and 2000 Hz. These frequencies contain fundamental frequencies of typical human speech and therefore can be used to quantify how well the particular surface will absorb sound including human voice in the space^38,43–45^. Accordingly, ${O}_{r}$ is calculated by adding up the equivalent sound absorption generated by all selected external materials (${OE}_{r}$) and internal materials (${OI}_{r}$) in room $r$, as shown in Eq. (5). (${OE}_{r}$) is calculated using the standard total equivalent sound absorption area per Sabine, which is summing up the product of multiplying the selected external material areas (${A}_{r,j}$) by their noise reduction coefficients $({a}_{{E}_{r,j,p}}$) and (${OI}_{r}$) is calculated similarly, as shown in Eq. (5). ${O}_{r}$ is then used in the standard Sabine formula in Eq. (6) to calculate the reverberation time ($R{T}_{60}^{r}$) for room $r$, which is required to calculate the minimum required level of sound absorption ($\ell_{r}$) using Eq. (7)^45^.

4 $${O}_{r}\ge \ell_{r}$$

5 $${O}_{r}={OE}_{r}+{OI}_{r}=\sum _{j=1}^{{J}_{r}}\sum _{p=1}^{P}{a}_{{E}_{r,j,p}}*{A}_{r,j}+\sum _{k=1}^{{K}_{r}}\sum _{f=1}^{F}{a}_{{I}_{r,k,f}}* {S}_{r,k}$$

6 $$R{T}_{60}^{r}=0.161* \frac{{V}_{r}}{{O}_{r}}$$

7 $$\ell_{r}= \frac{0.161 {V}_{r}}{R{T}_{60}^{r}}$$

where $\ell_{r}$ = total sound absorption coefficient required in room $r$ in sabine; ${O}_{r}$= total equivalent sound absorption area in room $r$ in Sabine/m^2^;${OE}_{r}$= equivalent sound absorption generated by the selected external acoustic materials;${OI}_{r}$= equivalent sound absorption generated by the selected internal acoustic materials;${a}_{{E}_{r,j,p}}$= noise reduction coefficient of used external acoustic material $p$ for surface $j$ in room $r$ in sabine; ${a}_{{I}_{r,k,f}}$= noise reduction coefficient of used internal acoustic material $f$ inside wall $k$ in room $r$ in sabine; ${V}_{r}$= volume of the room $r$ in cubic meter; $R{T}_{60}^{r}$= reverberation time in seconds per square meter for room $r$, where the sound be reduced by 60 dB in seconds and it is calculated using Eq. (6). For example, if a room required a total of 550 sound absorption in Sabine, the model is designed to make sure the acoustic materials selected will be capable of absorbing a minimum of 550 equivalent sound absorption in Sabine/m^2^ or more.

#### Speech intelligibility constraint

This constraint is formulated to ensure that the speech intelligibility requirement is compiled in each room. The speech intelligibility can be predicted using the percentage of Articulation Loss of Consonants (${\%AL}_{Cons}$) for room $r$ that can be calculated using Eq. (8) ^46^. Moreover, if ${D}_{r}$ ≥ ${D}_{L}$ the formula of (${\%AL}_{Cons}$) can be calculated, using Eq. (9), while ${D}_{L}$ calculated using Eq. (10). A recent study reported that speech intelligibility can be rated as (a) excellent when ${AL}_{Cons}$ is below 5%, (b) very good when ${AL}_{Cons}$ is between 5 and 10%, (c) good when ${AL}_{Cons}$ is between 10 and 15%, and (d) sufficient for only good listeners when ${AL}_{Cons}$ is above 15%, which is the limit for successful communication ^45,46^. Accordingly, the formulation of this constraint is designed to provide planners with the flexibility to specify their required rating of speech intelligibility in the office space rooms. Based on that designer-specified rating, the model can identify the required minimum ${S}_{min}$ and maximum ${S}_{max}$ level of speech intelligibility using the aforementioned rating scale, as shown in Eq. (13).

8 $${\%AL}_{cons}^{r}= \frac{656 {\left({D}_{r}\right)}^{2}{{(RT}_{60}^{r})}^{2}({n}_{r}+1)}{{V}_{r}{Q}_{r}{m}_{r}\overline{{a }_{r}}}+{G}_{r}$$

9 $${\%AL}_{cons}^{r}=9{ (RT}_{60}^{r})+{G}_{r}\quad if\; {D}_{r} \ge {D}_{L}$$

10 $${D}_{L}=3.16*0.141\sqrt{(Q)\ell_{r}}$$

11 $${m}_{r}=\frac{(1 - \overline{a })}{(1 - ac)}$$

12 $$\overline{a }=\frac{\sum_{i=1}^{n}{s}_{i}{ a}_{i}}{S}$$

13 $${S}_{min}< {\%AL}_{cons}^{r}\le {S}_{max}$$

where ${\mathrm{\%}AL}_{cons}^{r}$= the percentage of articulation loss of consonants for room $r$; ${D}_{r}$ = the distance from the sound source to the farthest listener in room $r$; ${n}_{r}$ = number of sound sources in room $r$; ${Q}_{r}$ = sound directivity factor for sound sources in room $r$, for example a live talker assumed to have *Q* = 2.5 at 2 kHz octave band; ${D}_{L}$= limited distance with no loss of ${AL}_{Cons}$; ${m}_{r}$= critical distance modifier that is calculated using Eq. (11); $\overline{a }$ = average absorption coefficient for room $r$ that is calculated using Eq. (12); $ac$ = the absorption in the area covered by the sound sources, in this case we consider it to be ${O}_{r}$; ${G}_{r}$ = the listener-talker correction constant for room $r$, typically good listener-talker can be as low as 1–3% while the worst can go up to 12.5%; ${S}_{min}$= minimum designer-specified value for %${AL}_{cons}^{r}$; and ${S}_{max}$= maximum designer-specified value for %${AL}_{cons}^{r}$.

#### Noise reduction constraint

This constraint is formulated to ensure that the internal acoustic materials installed in common walls in office spaces provide sufficient reduction in the noise transmitted between adjoining rooms. It should be noted that internal acoustic materials installed in wall cavities reduce sound transmission between adjoining rooms, however they do not affect the reverberation time of either the source or receiving rooms. Accordingly, this constraint is designed to ensure that the noise reduction (${NR}_{st}$) level of a common wall between source room ($s$) and receiving room ($t$) is greater than or equal a designer-specified level ($Y$), as shown in Eq. (14). This noise reduction (${NR}_{st}$) of a common wall represents the sound difference in both sides of the wall in dB and can be calculated using standard formulation, as shown in Eq. (15) ^30^. Noise reduction of common walls is an important acoustic design metric that is often used to estimate and control noise levels for adjoining rooms ^47^.

14 $${NR}_{st}\ge Y$$

15 $${NR}_{st}={STC}_{st}+10\mathit{log}\left(\frac{{O}_{t}}{{S}_{st}}\right)$$

where ${NR}_{st}$= noise reduction of a common wall between source room $s$ and receiving room $t$;$Y$= designer-specified minimum ${NR}_{st}$ that must be achieved by all common walls in the building; ${STC}_{st}$= sound transmission class for common wall $st$ that has a minimum rating of 45 in 500 Hz octave for single or composite walls between offices and/or conference rooms ^14^; ${O}_{t}$= total sound absorption in receiver room in Sabin/${m}^{2}$; ${S}_{st}$= total area of common surface $st$ between source room and receiver room in ${m}^{2}$.

### Materials selection constraints

#### Area constraint

This set of constraints is designed to ensure that the selected optimal area of acoustic material for each floor ${A}_{r,1}$, ceiling ${A}_{r,2}$, and wall ${A}_{r,j}$ in each room is less than or equal its corresponding available area (${S}_{r,1}, {S}_{r,2}, {S}_{r,j}$) in room *r*, as shown in Eq. (16), respectively.

16 $${A}_{r,j}\le {S}_{r,j} \quad\forall \;r j=1\,\, floor,\quad j=2 ceiling,\quad and\; j\in \left\{3,\dots ,{J}_{r}\right\}\,\, for\,walls$$

#### Materials limit constraint

To ensure practicality, this set of constraints is integrated in the model to provide designers with the flexibility to limit the number of acoustic materials that can be used in the office space, as shown in Eqs. (17–20). Designers can use this constraint to restrict the maximum number of acoustic materials that can be selected by the model for floors, ceilings, and walls in the entire office space. For example, a designer can limit the maximum number of acoustic materials for floors to one or two in order to expedite its installation during the construction of projects that have tight schedules.

17 $$NEF\le SEF$$

18 $$NEC\le SEC$$

19 $$NEW\le SEW$$

20 $$NIW\le SIW$$

where $NEF$= total number of selected external acoustic materials for floors;$NEC$= total number of selected external acoustic materials for ceilings;$NEW$= total number of selected external acoustic materials for walls; $SEF$= designer-specified maximum number of allowed materials for floors; $SEC$= designer-specified maximum number of allowed materials for ceilings; $SEC$= designer-specified maximum number of allowed materials for walls;$NIW$= total number of internal acoustic materials selected for use inside wall cavities; $SIW$= designer-specified maximum allowed number of internal acoustic materials.

#### Binary constraint

This set of binary constraints represents that (1) each available external acoustic material $p$ can be either selected (${E}_{r,j, p}$ = 1) or not selected (${E}_{r,j, p}$ = 0) for each surface $j$ in room $r$, as shown in Eq. (21); and (2) each available internal acoustic material $f$ can be either selected (${I}_{r,k, f}$ = 1) or not selected (${I}_{r,k, f}$ = 0) for each wall $k$ in room $r$, as shown in Eq. (22). Furthermore, the summation of these designer decisions is constrained to be less than or equal 1 to ensure that no more than one material type can be selected for each external and internal wall, as shown in Eqs. (23) and (24), respectively.

21 $${E}_{r,j, p}\in \left\{0, 1\right\} \quad\forall\; r,j,p$$

22 $${I}_{r,k,f}\in \left\{0, 1\right\} \quad\forall\; r,k, f$$

23 $$\sum_{p=1}^{P}{E}_{r,j,p}\le 1\quad \forall \;r,j$$

24 $$\sum_{f=1}^{F}{I}_{r,k,f}\le 1 \quad\forall \;r,k$$

## Model Implementation

**Figure 3:**
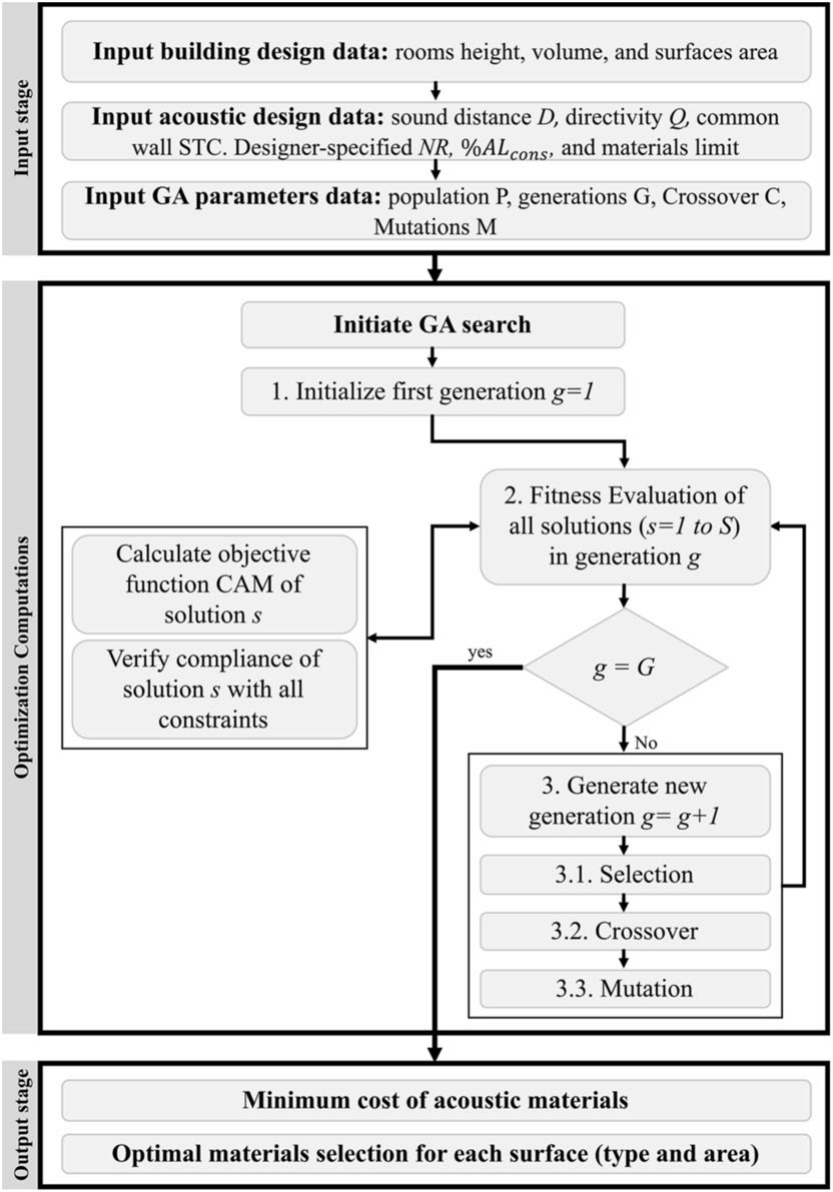
Model implementation using GA.

The developed model is implemented using genetic algorithms (GA) on MATLAB 2017b due to their capabilities of optimizing problems that includes mixed integer, nonlinear, discontinuous objective function, and constraints that are similar to those formulated in this model^48^. The model implementation is performed in three main stages: (1) input data, (2) optimization computations, and (3) output data, as shown in Fig. 3.

### Input data

The input stage enables designers to input all relevant building design data, acoustic design data, and parameters that are required to execute the optimization computations. The building design data should include all relevant room heights, volumes, and areas of all surfaces that can be readily obtained from the building design documents and/or BIM models. The acoustic design data includes sound distance (*D*), sound directivity (*Q*), sound transmission class (STC) for common walls, designer-specified noise reduction (NR) of common walls, the percentage of speech articulation of consonants (%${AL}_{Cons}$), the maximum number of materials that can be selected by the model, and all designer-specified feasible alternative materials and their thickness, Noise Reduction Coefficient (NRC) and cost per unit of measurement. The optimization parameters data that needs to be specified by designers include genetic algorithms population size (P), number of generations (G), crossover (C), and mutation rate (M), as shown in Fig. 3.

### Optimization computations

The model is designed to execute the optimization computations using genetic algorithms (GA). GA was used in the present model due to its reported capabilities of (a) exploring large search spaces, (b) solving convex, concave, mixed integer, and non-linear response function problems, and (c) analyzing both discrete and continuous objective functions ^49–53^. As shown in Fig. 3, the first step in the computation stage starts by creating an initial generation (*g* = 1) of randomly selected solutions (*s* = *1 to S*), where each solution represents one possible acoustic design for all rooms in the building. The model then evaluates the fitness of each solution (*s* = *1 to S*) in the current generation (*g*) (see step 2 in Fig. 3) by calculating its objective function cost of acoustic materials and verifying its compliance with all specified constraints. The model then examines if the total number of designer-specified generations was created and evaluated (*g* = *G*) and ends the optimization computations if this specified stopping condition was met. Otherwise, the model creates a new generation (*g* = *g* + 1) using GA selection, crossover, and mutation procedures and then repeats the aforementioned fitness evaluation step, as shown in step 3 in Fig. 3. The selection procedure (see step 3.1 in Fig. 3) starts by selecting pairs of solutions from the parent population that will be allowed to move to the reproduction phase to create a child population. This selection process is performed using a probabilistic approach that favors solutions with the least acoustic materials cost that was calculated earlier in the fitness evaluation step. Each pair of the selected population members is mated to produce new solutions in the population of the next generation (*g* = *g* + 1), using crossover and mutation operators. The crossover operation (see step 3.2 in Fig. 3) is performed to create a new child solution that contains a mix of the selected genetic information (i.e., acoustic material selections) of both parent solutions. This is achieved by randomly choosing a cutting point in the two strings of both parents, to swap a chunk of the genetic material from the first string with another chunk from the second. Newly generated child solutions are then mutated (see step 3.3 in Fig. 3) to randomly change the genetic information in the newly created solutions to maintain diversity in the population to prevent immature convergence to inferior solutions. Mutation probability is usually small to ensure that the mutation operation will not have a disruptive effect on the best members of the population ^54^. The fitness of the newly created solutions in the latest generation is then evaluated in step 2 of the optimization computations (see Fig. 3). These fitness evaluation and creation of new generations are iteratively repeated until the total number of designer-specified generations are evaluated, as shown in Fig. 3.

### Output data

**Figure 4:**
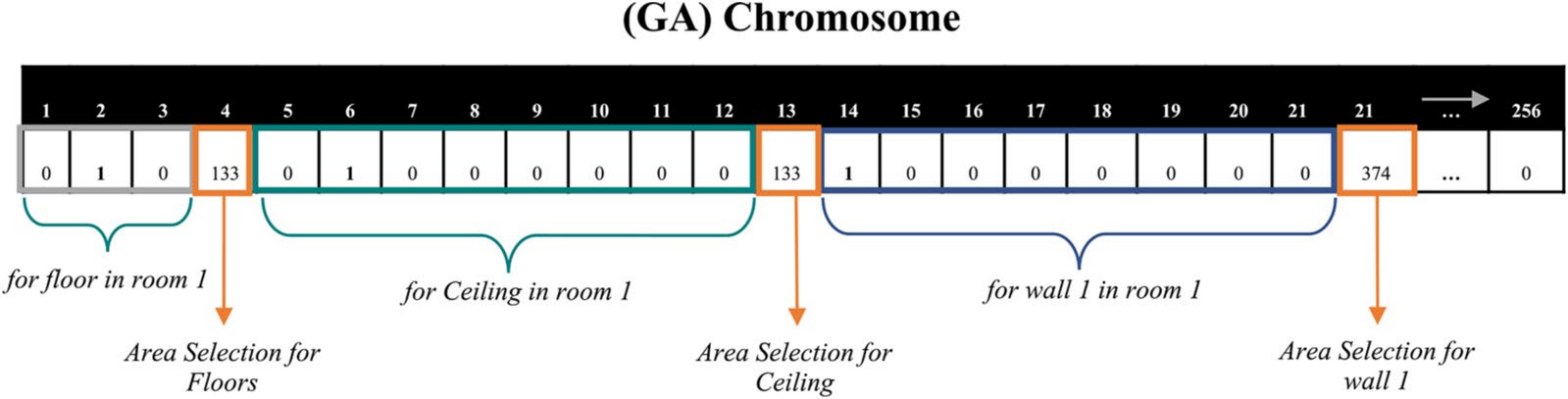
Example optimal materials type and area selection.

Upon the completion of the aforementioned optimization computations, the model generates a near-optimal solution that provides (1) the minimum total cost of acoustic materials that complies with all designer-specified acoustic requirements, and (2) the optimal selections of acoustic material type and area for each floor, ceiling, and or wall surface in the office space, as shown in Fig. 3. An example of these optimal selections of acoustic material type and area for each surface in office space is shown in Fig. 4.

The limitation of the developed model can be summarized, as follows: (1) neglecting three important acoustic parameters, sound source intensity, type, and its position that can affect the results accuracy; and (2) relaying mainly on the standard Sabine formula.

## Performance evaluation phase

**Figure 5:**
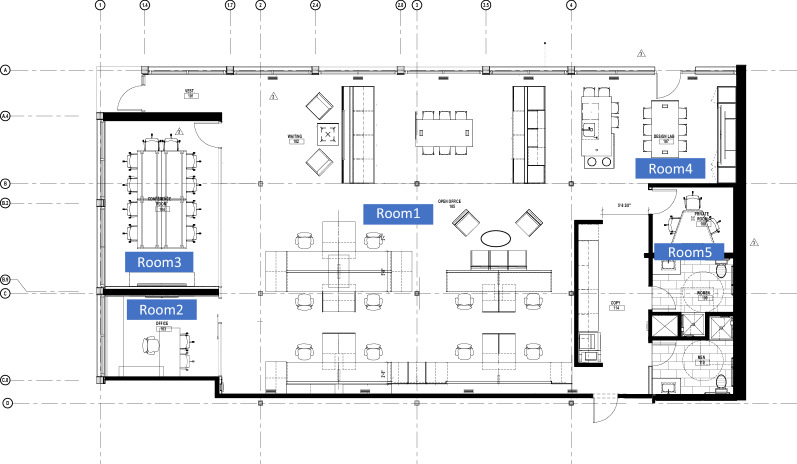
The first-floor layout design.

The purpose of this phase is to evaluate the performance of the developed model and demonstrate its novel and unique capabilities using a real-world application example. The example focuses on optimizing the acoustic design of the first floor of an office building that is currently under construction. The office space in the first floor has a total area of (324.8 m^2^) and it includes five main office spaces, including open floor plan cubical offices, private office, conference room, design lab, and small private meeting room, as shown in Fig. 5.

The acoustic design of this office space was optimized using the developed model in order to minimize the total cost of its acoustic material while satisfying all designer-specified acoustic quality requirements. The required input data for optimizing this example using the model includes: (1) building design data that was obtained from its design documents, as shown in Table 1; (2) acoustic design data and designer-specified level of noise reduction, percentage of articulation loss of consonants, and maximum number of acoustic materials that can be used in the office space, as shown in Tables 2 and 3; (3) feasible acoustic material alternatives data and their applicable surface, noise reduction coefficient, and cost per unit of measurement, as shown in Table 4; and (4) GA parameters data that were specified for this example to be a population size of 200, number of generations of 50,000, a crossover rate of 0.8, and mutation rate of 1.0. In this example, the model was used to identify the optimal selection of acoustic material type from 25 commercially available types for floors, ceilings, and walls. This creates an optimization problem with 256 designer decisions.

**Table 1:** Building design input data.

Rooms	Height m	Volume m^3^	Surface area m^2^					
			Floor	Ceiling	Wall 1	Wall 2	Wall 3	Wall 4
1	2.7	340	124	124	7	6	N/A	N/A
2		34	13	13	12	12	N/A	N/A
3		75.6	297	297	132	132	N/A	N/A
4		84.6	31	31	13	185	N/A	N/A
5		22	8	8	9	9	10	10

**Table 2:** Acoustic design input data.

Rooms	Design requirements			Designer-specified	
	Sound distance (D)	Sound directivity (Q)	Sound transmission class (STC)	Noise reduction in dB (NR)	Articulation loss of consonant (${\%AL}_{Cons}$)
1	12 m	8	45	45	10
2	3 m	4		45	10
3	6 m	4		50	5
4	6 m	4		45	10
5	2 m	4		50	10

**Table 3:** Maximum number of acoustic materials that can be used in the office space.

Type of material	Designer-specified maximum number of materials	
External	${\varvec{S}}{\varvec{E}}{\varvec{F}}$ *on floor*	2
	${\varvec{S}}{\varvec{E}}{\varvec{C}}$ *on ceiling*	2
Internal	${\varvec{S}}{\varvec{E}}{\varvec{W}}$ *on walls surfaces*	5
	${\varvec{S}}{\varvec{I}}{\varvec{W}}$ *inside walls cavity*	1

**Table 4:** Feasible alternatives of acoustic material.

No.	Acoustic material type	Applicable surface	Noise Reduction Coefficient (NRC)	Cost/m^2^ ($)
1	Class A Anechoic Studio Foam	Walls and Ceilings	0.8	103.23
2	Soundproofing Insulation	Behind Walls and Ceilings	0.95	23.04
3	Standard Fabric Wrapped Acoustic Panels	Walls and Ceilings	0.85	106.35
4	Art digital printed panel	Walls	0.85	322.93
5	Acoustic Partition	Floors	1	269.11
6	HARMONI Acoustic Ceiling Tiles	Ceilings	0.95	107.64
7	Perforated Wood Art Panels	Floors and Ceiling	0.85	150.70
8	Flooring underlayment (Low Duty)	Floors	1.2	63.51
9	AcoustiTherm Acoustic Ceiling Tiles (3")	Ceilings	1.05	44.99
10	Acoustic Ceiling Tiles (2") "A"	Ceilings	0.55	16.15
11	CrossPoint Sound Absorbing Fabrics	Walls	0.2	19.38
12	Sonex® Audio Tiles (1")	Walls and Ceilings	0.5	40.36
13	Sonex® One acoustic foam panels (2")	Walls and Ceilings	0.85	62.43
14	Sonex® One acoustic foam panels (3")	Walls and Ceilings	1.05	94.73
15	Signature Sound Barrier Ceiling Tile	Ceilings	0.85	309.47
16	HVAC and Ceiling Sound Barriers	Ceilings	1	331.54
17	Acoustic Ceiling Tiles "B"	Ceilings	0.85	0.65
18	AudioSeal® Combination Sound Blanket	Walls	0.75	107.64
19	Cork flooring	Floorings	1	10.76
20	Acoustic Foam	Ceilings	0.75	333.69
21	Ceiling Sonex® Rondo Sound Baffles	Ceilings	0.75	287.94
22	Roxul Rock board 80–2 inches’	Walls	0.9	172.22
23	Guilford of Maine Acoustic Fabric	Walls	0.05	41.98
24	Auralex StudioFoamPro	Floors	0.9	80.73
25	Install Carpet	Floors	0.40	4.84

The optimization computations for this example were performed using a personal laptop with Intel Core i5 2.8 GHz, and 8 GB RAM, and its elapsed computational time was one hour and 47 min. The aforementioned design input data was analyzed by the developed model to search for and identify the near-optimal solution for each of the 256 designer decisions in this application example (see sample results in Table 5). This optimal solution produced a minimum total cost of $13,545 for the acoustic materials that are needed to fully comply with all the specified acoustic quality constraints in this example (see Table 3). The generated optimal solution for this example includes (1) the optimal selections for the type and area of acoustic material for each surface in all the rooms in the office space, and (2) the achieved acoustic quality performance by the selected materials for each room, including noise reduction (*NR*_*st*_) level for common walls, reverberation time ($$R{T}_{60}^{r}$$), total equivalent sound absorption (*O*_*r*_) in Sabine/m^2^, and articulation loss of consonant ($${\%AL}_{Cons}$$), as shown in the sample results in Table 5. For example, the model provided the following optimal selections for the conference room: (1) cover the entire external surface area of the floor and ceiling (28 m^2^) with ‘Cork flooring’ and ‘HARMONI Acoustic Ceiling Tiles’, respectively; (2) install (13 m^2^) of ‘Guilford of Maine Acoustic Fabric’ on the external surface of wall 1; (3) do not install any acoustic materials on the external surface of wall 2; (5) install (13 m^2^) of ‘Roxul Rock board 80–2 inches’ in the internal cavity of wall 1; and (6) do not install any acoustic materials in the internal cavity of wall 2. Similarly, the model provided optimal acoustic material type and area selections for the remaining rooms in this example, as shown in the sample results in Table 5.

**Table 5:** Sample optimal acoustic material selections and achieved acoustic quality for three rooms.

Rooms	Acoustic material placement	Surface	Optimal acoustic material selection			Achieved acoustic quality			
			Type	Area m^2^	Cost $/m^2^	*NR*_*st*_ dB	$$R{T}_{60}^{r}$$	*O*_*r*_ Sabine/m^2^	$${\mathrm{\%}AL}_{cons}^{r}$$
**(1) Open floor plan**	External Surfaces	floor	Install Carpet	124	$4.84		*0.68*	*940.57*	*6%*
		ceiling	Acoustic Ceiling Tiles (2") "A"	124	$16.15				
		wall 1	Sonex® Audio Tiles (1")	7	$40.36				
		wall 2	Sonex® Audio Tiles (1")	6	$40.36				
		wall 3	none	0	$0.00				
	Internal Wall Cavities	wall 1	none	0	$0.00	–			
		wall 2	none	0	$0.00	–			
**(2) Private office**	External Surfaces	floor	Cork flooring	13	$10.76		*0.86*	*147.4*	*7.7%*
		ceiling	HARMONI Acoustic Ceiling Tiles	13	$107.64				
		wall 1	none	0	$0.00				
		wall 2	none	0	$0.00				
	Internal Wall Cavities	wall 1	none	0	$0.00	–			
		wall 2	none	0	$0.00	–			
**(3) Conference room**	External Surfaces	floor	Cork flooring	28	$10.76		*0.64*	*432*	*5%*
		ceiling	HARMONI Acoustic Ceiling Tiles	28	$107.64				
		wall 1	Guilford of Maine Acoustic Fabric	13	$41.98				
		wall 2	none	0	$0.00				
	Internal Wall Cavities	wall 1	Roxul Rock board 80-2 inches	13	$172.22	50			
		Wall 2	none	0	$0.00	–			

A comparative analysis was conducted to compare the minimum acoustic material cost generated by the model to cost estimates provided by several acoustic design firms and websites for the analyzed real-world example^55–57^. The provided cost estimates of acoustic materials for this example by these firms ranged from $15,000 and $25,000 which is significantly higher than the $13,545 total cost of acoustic materials provided by the model. This illustrates the novel capability of the developed model in minimizing the total cost of acoustic materials for real-world office spaces while satisfying all designer-specified acoustic quality requirements.

## Summary and conclusions

This paper presented the development of an original optimization model that is capable of supporting designers, architects, and decision-makers in identifying optimal acoustic material selection and design for office spaces. The model is capable of achieving all designer-specified acoustic quality requirements while minimizing the cost of acoustic materials for office space. The model was developed in five main stages that focused on (1) identifying all relevant designer decisions that have an impact on the building acoustic quality and cost; (2) formulating an the model objective function that is capable of minimizing the cost of acoustic materials; (3) identifying all practical model constraints; (4) implementing the model using genetic algorithms (GA); and (5) evaluating the model performance using a real-world application example of an office space design. The evaluation of this real-world application example illustrates the novel and unique capability of the model in searching all feasible acoustic material types and providing an optimal selection of material type and area for each floor, ceiling, and wall in office spaces. These optimal selections enable designers to minimize the total cost of acoustic material of office spaces while achieving all designer-specified acoustic quality requirements. Future expansions of the model include (1) integrating sound source intensity, type and its position parameters to explore their influences and contributions in evaluating and calculating acoustic quality in office spaces; (2) considering related acoustic simulation software to evaluate acoustic characteristics and sound field that will capture the sound frequency changes; (3) testing the acoustic quality on site with a real situation to compare the actual acoustic quality with the results calculated using standards formulas.

## Abbreviations

CAM: Total cost of acoustic materials
CEM: Total cost of all external acoustic materials
*C*_*P*_: Unit cost of external acoustic material *p* in $/m^2^
*E*_*r,j,p*_: Binary decision variable representing the selection of external acoustic material *p* for surface *j* in room *r*
*A*_*r,j*_: Total selected acoustic material area for surface *j* in room *r* in m^2^
CIM: Total cost of all internal acoustic materials
*C*_*f*_: Unit cost of internal acoustic material *f* in $/m^2^
*I*_*r,k,f*_: Binary decision variable representing the selection of internal acoustic material *f* used inside wall *k* in room *r*
*R*: Total number of rooms in the building
*J*_*r*_: Total number of wall surfaces in room *r*
*P*: Total number of external acoustic materials that can be installed on floor, ceiling, and walls
*K*_*r*_: Total number of walls in room *r*
*S*_*r,k*_: Total area of wall *k* in room *r* in m^2^
*F*: Total number of internal acoustic materials that can be installed inside walls cavity
$$\ell_{r}$$: Total sound absorption coefficient required in room *r*
*O*_*r*_: Total sound absorption in room *r*
*OE*_*r*_: Equivalent sound absorption generated by the selected external acoustic materials
*OI*_*r*_: Equivalent sound absorption generated by the selected internal acoustic materials
$${a}_{{E}_{r,j,p}}$$: Sound absorption coefficient of used external acoustic material *p* for surface *j* in room *r*
$${a}_{{I}_{r,k,f}}$$: Sound absorption coefficient of used internal acoustic material *f* inside wall *k* in room *r*
*V*_*r*_: Volume of the room *r* in cubic meter
$$R{T}_{60}^{r}$$: Reverberation time in seconds per square meter for room *r*
$${AL}_{cons}^{r}$$: Articulation loss of consonants for room *r*
*D*_*r*_: The distance from the sound source to the farthest listener in room *r*
*n*_*r*_: Number of sound sources in room *r*
*Q*_*r*_: Sound directivity factor for sound sources in room *r*
*D*_*L*_: Limited distance with no loss of *AL*_*Cons*_
*m*_*r*_: Critical distance modifier
$$\overline{a }$$: Average absorption coefficient for room *r*
*ac*: The absorption in the area covered by the sound sources
*G*_*r*_: The listener-talker correction constant for room *r*
*S*_min_: Minimum designer-specified value for %*AL*_*cons*_^*r*^
*S*_max_: Maximum designer-specified value for %*AL*_*cons*_^*r*^
*NR*_*st*_: Noise reduction of a common wall between source room *s* and receiving room *t*
*Y*: Designer-specified minimum *NR*_*st*_ that must be achieved by all common walls in the building
*STC*_*st*_: Sound transmission class for common wall *st*
*NEF*: Total number of selected external acoustic materials for floors
*NEC*: Total number of selected external acoustic materials for ceilings
*NEW*: Total number of selected external acoustic materials for walls
*SEF*: Designer-specified maximum number of allowed materials for floors
*SEC*: Designer-specified maximum number of allowed materials for ceilings
*SEC*: Designer-specified maximum number of allowed materials for walls
*NIW*: Total number of internal acoustic materials selected for use inside wall cavities
*SIW*: Designer-specified maximum allowed number of internal acoustic materials
